# Relationships between training load, peak height velocity, muscle soreness and fatigue status in elite-level young soccer players: a competition season study

**DOI:** 10.1186/s12887-023-03869-7

**Published:** 2023-02-03

**Authors:** Hadi Nobari, Özgür Eken, Okan Kamiş, Rafael Oliveira, Pablo Prieto González, Rodrigo Aquino

**Affiliations:** 1 Department of Exercise Physiology, Faculty of Educational Sciences and Psychology, University of Mohaghegh Ardabili, Ardabil, 56199-11367 Iran; 2 Department of Motor Performance, Faculty of Physical Education and Mountain Sports, Transilvania University of Braşov, 500068 Braşov, Romania; 3 Faculty of Sports Sciences, University of Extremadura, 10003 Cáceres, Spain; 4 Department of Physical Education and Sport Teaching, Inonu University, Malatya, Turkey; 5 Faculty of Sports Sciences, Aksaray University, 68100 Aksaray, Türkiye; 6 Sports Science School of Rio Maior—Polytechnic Institute of Santarém, 2040-413 Rio Maior, Portugal; 7 Research Center in Sport Sciences, Health Sciences and Human Development, 5001-801 Vila Real, Portugal; 8 Life Quality Research Centre, 2040-413 Rio Maior, Portugal; 9 Health and Physical Education Department, Prince Sultan University, Riyadh, 11586 Saudi Arabia; 10 Lab Sport, Department of Sports, Centre of Physical Education and Sports, Federal University of Espírito Santo, Vitória, 29075810 Brazil

**Keywords:** Internal load, Football association, Sports sciences, Youth, Acute load, Chronic load

## Abstract

**Background:**

This study aimed to compare training load parameters, delayed onset muscle soreness (DOMS), and fatigue status between season periods (1^st^ and 2^nd^ halves) in U14 soccer players and to analyze the relationships between training load parameters based on season periods (1^st^ and 2^nd^ halves) with peak height velocity (PHV), DOMS, and fatigue status in under-14 (U14) young elite soccer players. Additionally, it was intended to analyze if fatigue, DOMS and PHV could explain training load parameters across the season.

**Methods:**

Twenty U14 players that competed in the national league participated in this study. The players were monitored during the whole season (26 weeks), and evaluations were carried out at the end of the in-season. Anthropometric and body composition parameters and the maturity offset of each player were utilized to compute each player's age at PHV. Players reported their levels of DOMS and fatigue status using Hooper index questionnaires. The internal load was monitored using the rating of perceived exertion (RPE). Acute weekly internal load (AW), chronic weekly internal load (CW), acute: chronic workload ratio (ACWR), training monotony (TM), and training strain (TS) were also obtained.

**Results:**

The main results showed that TM was higher in the 2^nd^ half, while CW, AW and DOMS were higher in the 1^st^ half of the season. Moreover, the main correlations showed a positive correlation between PHV and TS (2^nd^ half of the season) and between fatigue and TM (1^st^ half of the season).

**Conclusion:**

In conclusion, variations in well-being status and PHV cannot explain the variations in internal training loads in elite U14 soccer players. In addition, internal training load indices during the first half of the competitive season can promote a fundamental base for progression loads during the second period of the competitive season.

## Introduction

Team sports players compete monthly or biweekly, necessitating more frequent and time-consuming choices about player health and weariness. Under such conditions, the most effective monitoring methods may be those responsive to dramatic load fluctuations [1]. Understanding individual responses to training, monitoring fatigue recovery, and avoiding overtraining and injury are all made possible by closely monitoring an athlete's training load [2].

Training loads can be generally classified as "internal" or "external" workloads [3]. Internal workloads are a measure of the athlete's perceived level of effort (e.g., rating of perceived exertion or heart rate response to the stimulus), whereas external workloads are typically the quantification of an athlete's external workloads by a third party (e.g., running distance covered) [4].

The abovementioned facts highlight how crucial it is to evaluate internal load throughout various strength and conditioning training sessions and keep an eye on players' wellness levels to give athletes and coaches essential information [3]. The distribution of training sessions, game lengths, and these variables' links with well-being, particularly in young players, are poorly understood [5].

Significant correlations between delayed onset muscle soreness (DOMS), stress, tiredness perception, and sleep quality are presented in the research [4, 6, 7]. For instance, an earlier study found a correlation between DOMS and soccer players' training/match load over the season. Additionally, overall load and load variations can affect particular wellness traits like soreness and exhaustion in the neuromuscular system [8–10].

Previous studies investigated load monitoring among elite junior players [11, 12]. According to Nobari et al., there is a strong correlation between acute training load and measures of well-being, such as the Hooper index and its items (fatigue, stress, sleep quality and DOMS) [12]. These associations may offer valuable insight for sports scientists, coaches, or even strength and conditioning specialists to manage the training process effectively, acquire improvements, and prevent poor adaptations that can interfere with sleep quality, stress, and DOMS and thereby impair performance [13].

To the author's knowledge, various soccer studies have been published to examine the relationships between workload and maturity in U14 [14] and U16 players [15, 16]. However, little is known regarding the relationship among the training load, peak height velocity (PHV), muscle soreness and fatigue status in elite-level young soccer players who were recently recommended to be studied throughout the competition season [14, 17]. Therefore, this study has the potential to bridge the gap between science and practice.

In sum, this study compared training load parameters, DOMS and fatigue status between season periods (1^st^ and 2^nd^ halves) in U14 soccer players. In addition, it analyzed the relationships between training load parameters based on season periods (1^st^ and 2^nd^ halves) with PHV, DOMS and fatigue status. Additionally, it was intended to analyze if training load parameters could explain fatigue, DOMS, and PHV.

## Materials and methods

### Participants

The t-test family sample power was calculated a priori to compute achieved power: α error prob level = 0.05; effect size = 0.7; 1 − β error prob = 0.8 by the G*Power tool. There was an 80% actual power with the present analysis of 19 subjects [18]. We conducted this analysis with G*Power software (University of Düsseldorf, Düsseldorf, Germany). Twenty elite young players (mean ± standard deviation (SD); chronological age: 13.3 ± 0.5 years; height: 165.8 ± 11.7 cm; body mass: 50.7 ± 7.6 kg; peak height velocity: 13.3 ± 0.2 years; maturity off-set: -0.01 ± 0.56 years), who regularly participated in football training, participated in this study's sample. These individuals competed in the U14 age bracket and, following the program established by the relevant federation, they first participated in the regional league and then progressed to the national league. The team had three attackers, four central defenders, four central midfielders, four wide defenders, five wide midfielders, and four central defenders. The inclusion criteria were as follows: 1) at least three years of soccer experience; 2) active and regular participation in all phases of the study; 3) participants were not permitted to use any growth or maturation-affecting supplements; and 4) participants were not permitted to perform additional exercises. Exclusion criteria included: 1) not participating in 80% of competitions (formal and informal) and training sessions during the season; 2) not attending one of the study's medical or physical examinations. The University of Mohaghegh Ardabili Ethical Committee approved this research. Similarly, we have done so with the Helsinki declaration (2013). All participants were informed of the risks and benefits of this study. They have the option to withdraw at any time. The informed consent form was signed by the parent /legal guardian and players at the beginning of the study.

### Study design

**Fig. 1:**
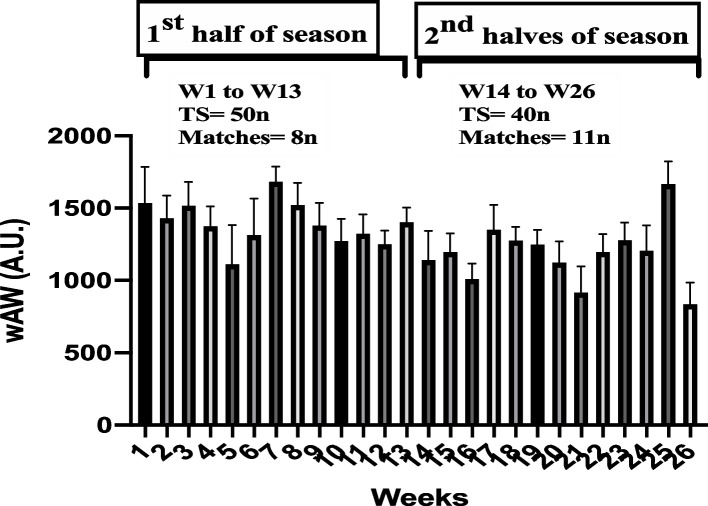
Timeline of monitoring on weekly acute workload (wAW) in the entire study. W, week; TS, training sessions

This investigation was carried out as a prospective study using an observational cohort design. Researchers monitored the players during the whole season, and evaluations were carried out once the season containing the competitive matches had concluded. The current investigation was carried out over the course of 26 weeks. We divided the entire season into two halves equal in length (1^st^ and 2^nd^ halves). The season was split into two halves: the first half (July to October, weeks 1 to 13 (8 matches and 50 training sessions (TS)) and the second halves (October to January, weeks 14 to26 weeks (11 games and 40 TS)) (Fig. 1).

Anthropometric parameters and body composition of the players were measured in one day. Then we calculated the maturity offset of each player. Players reported their levels of DOMS and fatigue status using Hooper index questioners (~ 30 min before the sessions) [19]. In addition, the RPE was monitored at the end of each training session (~ 30 min). One week in advance of the evaluation, there was a familiarization session that was planned. This "training load" was then calculated in conjunction with the total training time to determine the total amount of accumulated effort for any given period.

### Anthropometric measures and maturity offset

All anthropometric measurements, as well as measurements of body composition, were taken first thing in the morning. A skilled person used a stadiometer (Seca model 213, Germany) to measure the subject's height and sitting height with an accuracy of 5 mm. The subject's weight was measured and recorded using a digital scale (Seca model 813, UK) with a precision of 0.1 per kg. The maturity offset and age at PHV were established by applying the Mirwald algorithm to the data acquired up top and basing the results on the collected information (16). Based on the information contained above and using the Mirwald formula, the maturity offset and age at PHV were determined [20]. The procedure used is as follows: maturity offset =  − 9.236 + (0.0002708 X (leg length × sitting height)) + (- 0.001663 X (age × leg length)) + (0.007216 (age × sitting height)) + (0.02292 X (Mass by stature ratio X 100)), where *R* = 0.94, R2 = 0.891, and SEE = 0.592) and for leg length = standing height (cm)—sitting height (cm) was used and PHV = Age at measurement—maturity offset. The athlete's time away from PHV is reflected in the maturity offset. The athlete has not yet attained PHV if the offset is negative. Positive offsets show that PHV has already taken place. Based on the aim of the study, only PHV was used to address the purposes of the study.

### Internal training load

Each player was asked, "How did you feel about the intensity of the training?” for each session on a Category-Ratio-10 Borg scale, half an hour after training. In this scale, number one refers to a short training session, and ten refers to a very high-intensity training session [21]. This scale was translated for Iranian players to provide their answers for better clarity. The translation is not validated, but it was made by a professional in both Iranian and English.

Then, WL was calculated considering s-RPE and training time for each training session. These data were used to obtain information and analyze weekly workload parameters (AW = the accumulated acute workload in the season; CW = the accumulated chronic workload in the season; ACWLR = the accumulated acute: chronic workload ratio in the season; TM = the accumulated training monotony in the season; TS = the accumulated training strain in the season) [22]. Thus, the following calculations were made: [22–25]

$$ACWLR=acute\;workload(most\;recent\;week)/chronic\;workload(last\;4\;weeks)$$

$$TM=mean\;training\;load\;during\;the\;seven\;days\;of\;the\;week/standard\;deviation\;of\;training\;load\;during\;the\;seven\;days\;of\;the\;week$$

$$TS=sum\;of\;the\;training\;loads\;for\;all\;weekly\;sessions\times TM$$

Considering that 26 weeks were analysed, the average for all 26 weeks was used to provide the final value of AW, CW, ACWLR, TM and TS.

In addition, workload, DOMS and fatigue parameters are shown with abbreviations such as AW1, AW2, CW1, CW2, ACWLR1, ACWLR2, TM1, TM2, TS1 and TS2 for the 1st and 2nd halves of the season. Besides, all training load parameters are shown with abbreviations such as AW-total, CW- total, ACWLR- total, TM- total, TS- total.

### Well-being status

In this study, we aimed to consider fatigue and DOMS. For that reason, Hopper Index questionnaire [19] used to gather data (e.g., scale of 1–7, in which 1 is very, very low and 7 is very, very high). This questionnaire was taken into consideration half an hour before the start of each session. Before beginning the study, the participants were given instructions on how to use the scale. The data mentioned above were obtained by adding up the values of each variable over a week. It was decided to collect data independently so that the players wouldn't overhear the results of their teammates' competitions. Excel was the program of choice for developing the daily data register.

For better clarity, both fatigue and DOMS of Hooper index were translated for Iranian to players to provide their answers. The translation is not validated, but it was made by a professional in both Iranian and English. This questionnaire has been used since four years ago, from the first studies by Nobari et al. [13] in Iran.

### Statistical analysis

Statistical analyses were performed using GraphPad Prism 8.0.1 (GraphPad Software Inc, San Diego, California, USA). The significance level was set at *p* < 0.05. Normality assumptions of the data were determined by Shapiro–Wilk test, skewness and kurtosis values. Since the data showed normal distribution, the variables were summarized as mean ± standard deviation (SD). Paired Samples t-Test was used to compare training load parameters (AW, CW, ACWLR, TM1, and TM, TS), fatigue and DOMS values according to season periods (1^st^ and 2^nd^ phases). Cohen's d effect sizes were calculated and expressed with a 95% confidence interval to document the size of the observed statistical effects. These effect sizes were defined as 0.2 = trivial, 0.2 to 0.6 = small effect, 0.6 to 1.2 = moderate effect, 1.2 to 2.0 = large effect and 2.0 = very large [26]. Pearson correlation analysis was performed between training load parameters (AW1, AW2, CW1, CW2, ACWLR1, ACWLR2, TM1, TM2, TS1 and TS2) periods using PHV, fatigue-1, fatigue-2, DOMS-1, DOMS-2 factors and training load parameters (AW-total, CW- total, ACWLR- total, TM- total, TS- total) periods using PHV, fatigue, DOMS factors. The following ranges were considered for the correlation coefficient sizes: < 0.1 = trivial; 0.1–0.3 = small; > 0.3–0.5 = moderate; > 0.5–0.7 = large; > 0.7–0.9 = very large; and > 0.9 = nearly perfect [27]. Multiple linear regression has been analyzed between workload parameters with maturation variables, DOMS, and fatigue. The significance level was considered at *p* ≤ 0.05.

## Results

**Fig. 2:**
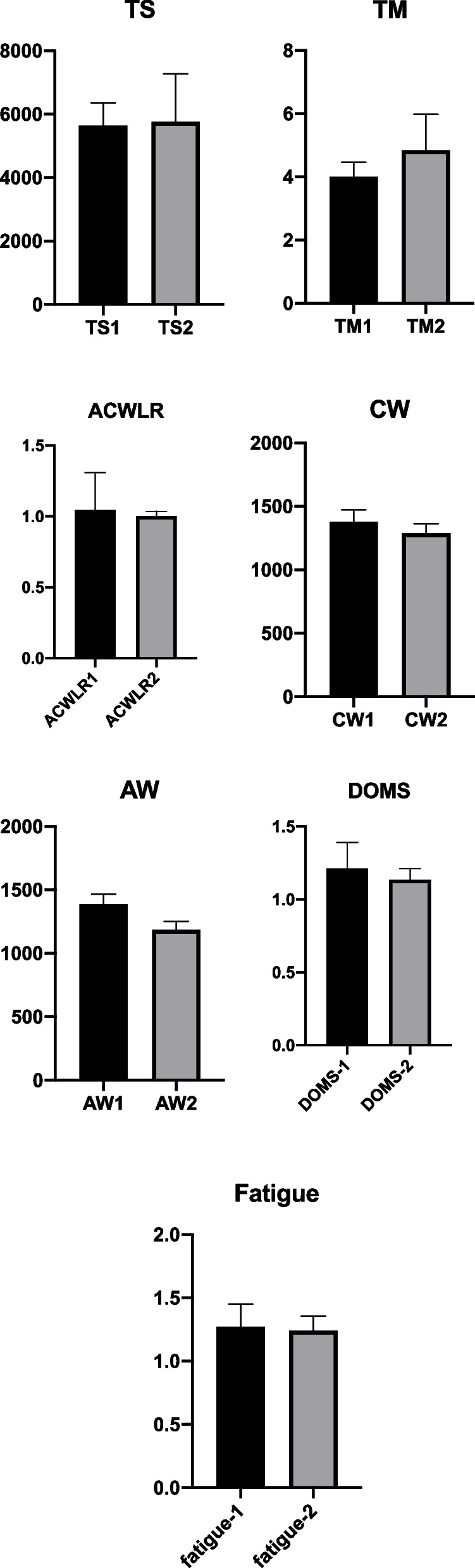
Comparison of training load parameters based on season periods (1^st^ and 2^nd^ phases), delayed onset muscle soreness (DOMS) and fatigue status. AW = the accumulated acute workload in the season; CW = the accumulated chronic workload in the season; ACWLR = the accumulated acute: chronic workload ration in the season; TM = the accumulated training monotony in the season; TS = the accumulated training strain in the season; 1 = 1st half of season; 2nd halves of the season

Figure 2 presents training load parameters based on season periods (1^st^ and 2^nd^ phases), delayed onset muscle soreness (DOMS) and fatigue status. TM (*p* = 0.004; ES = -0.72) was statistically significant based on season periods (1^st^ and 2^nd^ phases). CW (*p* < 0.001; ES = 2.34) was statistically significant based on season periods (1^st^ and 2^nd^ phases). AW (*p* < 0.001; ES = 2.88) was statistically significant based on season periods (1^st^ and 2^nd^ phases). DOMS (*p* = 0.041; ES = 0.49) was statistically significant based on season periods (1^st^ and 2^nd^ phases).

Some variables did not statistically significant based on season periods (1^st^ and 2^nd^ phases). TS (*p* = 0.725; ES = -0.08), ACWLR (*p* = 0.491; ES = 0.157), and fatigue (*p* = 0.475; ES = 0.163) were not statistically significant based on season periods (1^st^ and 2^nd^ phases).

Table 1 shows the analysis of the correlation between training load parameters (AW, CW, ACWLR, TS, and TM) based on periods (1^st^ and 2^nd^ halves of the season) with PHV DOMS and fatigue variables.

**Table 1:** Analysis of correlation between training loads parameters (AW, CW, ACWLR, TS, and TM) based on periods (1^st^ and 2^nd^ halves of season) with PHV, fatigue and DOMS variable

**Variable**	PHV	Fatigue-1	Fatigue-2	DOMS-1	DOMS-2	AW1	AW2	CW1	CW2	ACWLR1	ACWLR2	TM1	TM2	TS1	TS2
PHV	—														
Fatigue-1	-0.326	—													
Fatigue-2	-0.051	0.304	—												
DOMS-1	-0.426	0.501	0.140	—											
DOMS-2	-0.242	-0.037	0.448	0.488	—										
AW1	-0.057	-0.053	-0.024	0.103	0.281	—									
AW2	0.360	-0.297	0.125	-0.166	0.228	0.579	—								
CW1	-0.127	-0.064	-0.058	0.063	0.246	0.981	0.571	—							
CW2	0.066	-0.176	0.017	-0.033	0.262	0.926	0.826	0.933	—						
ACWLR1	-0.227	4.476	-0.076	-0.060	0.032	-0.258	-0.385	-0.268	-0.347	—					
ACWLR2	0.164	-0.066	0.032	0.033	0.143	-0.059	0.030	-0.095	-0.073	-0.110	—				
TM1	-0.300	**-0.468**	-0.357	-0.116	0.070	0.010	-0.151	0.043	-0.047	0.639	0.247	—			
TM2	0.421	-0.265	-0.130	-0.278	0.015	-0.222	0.137	-0.275	-0.137	0.116	0.609	0.209	—		
TS1	-0.235	-0.158	-0.335	-0.058	0.207	0.501	0.202	0.529	0.440	0.339	0.262	0.855	0.100	—	
TS2	**0.479**	-0.295	-0.111	-0.290	0.017	-0.066	0.366	-0.118	0.072	-0.012	0.571	0.123	0.966	0.111	—

*PHV* Peak height velocity, *AW* Acute workload, *CW* Chronic workload, *ACWLR* Acute: chronic workload ration, *TM* Training monotony, *TS* Training strain, 1 = 1^st^ half of season and 2 = 2^nd^ half of season, respectively

Significant differences (*p* ≤ 0.05) are highlighted in bold

In the correlations between training loads parameters based on periods with PHV, DOMS and fatigue variables, the results were: PHV to TS in the second half (*r* = 0.479 moderate, CI 95% {0.760 to 0.046}; *p* = 0.033) are largely related. Fatigue in the second half to TM in the first half (*r* = -0.468 large, CI 95% {-0.760 to -0.018}; *p* = 0.037) are moderately related. DOMS in the first half and DOMS in the second half (*r* = 0.488, CI 95% {0.045 to 0.771}; *p* = 0.028) are moderately related.

Table 2 shows the analysis of the correlation between training load parameters (AW-Total, CW- Total, ACWLR- Total, TS- Total, and TM- Total) based on PHV and wellness variables in which the following were noted: AW total to CW total (*r* = 0.930, CI 95% {0.972 to 0.829}; *p* < 0.001; nearly perfect correlated); ACWLR total and CW total (*r* = -0.621, CI 95% {-0.246 to -0.0834}; *p* = 0.003; large correlated); TS total to TM total (*r* = 0.892 CI 95% {0.957 to 0.743}; *p* < 0.001; nearly perfect correlated).

**Table 2:** Analysis of correlation between the total training load parameters based on maturation and wellness variables

Pearson’s Correlations								
**Variables**	PHV	Fatiguetotal	DOMStotal	AWtotal	CWtotal	ACWLR-Total	TMtotal	TStotal
PHV	—							
Fatiguetotal	-0.270	—						
DOMStotal	-0.427	0.419	—					
AWtotal	0.160	-0.103	0.088	—				
CWtotal	0.085	-0.069	0.037	**0.930**	—			
ACWLR-Total	-0.233	-0.006	-0.029	-0.432	**-0.621**	—		
TMtotal	0.424	-0.353	-0.316	0.048	-0.101	0.176	—	
TStotal	0.439	-0.352	-0.253	0.480	0.326	-0.060	**0.892**	—

*PHV* Peak height velocity, *AW-Total* Total amount of acute workload, *CW-Total* Total amount of chronic workload, *ACWLR-Total* Total amount of acute: chronic workload ratio, *TM-Total* Total amount of training monotony, *TS-Total* Total amount of training strain

Significant differences (*p* ≤ 0.05) are highlighted in bold

A multilinear regression model was used to determine the independent predictors of training load parameters (AW, CW, ACWLR, TS, and TM) durations using PHV, DOMS and fatigue factors. However, their coefficients were not determined to be statistically significant (*p* > 0.05; Table 3).

**Table 3:** Multiple linear regression analysis: Percentage of variation between training load parameters with maturity, DOMS and fatigue variables

**Variable**	**Beta**	**Estimate**	|**t**|	***p* Value**	**95% CI for Estimated**	
**AW**	β0	821.5	1.384	0.18	-436.4 to 2079	***R***^2^ = 0.072 Adjusted ***R***^2^ = -0.10 ***p*** = 0.74 **AIC** = 180.6
Fatigue	β1	-80.89	0.54	0.59	-397.9 to 236.1	
DOMS	β2	149.6	0.85	0.40	-219.4 to 518.6	
PHV (years)	β3	29.33	0.84	0.41	-44.69 to 103.4	
**CW**	β0	1071	1.64	0.11	-308.6 to 2450	***R***^2^ = 0.04 Adjusted ***R***^2^ = -0.13 ***p*** = 0.87 **AIC** = 184.3
Fatigue	β1	-105.5	0.64	0.52	-453.1 to 242.1	
DOMS	β2	117.2	0.61	0.54	-287.5 to 521.8	
PHV (years)	β3	15.60	0.40	0.68	-65.57 to 96.78	
**ACWLR**	β0	2.02	2.14	0.04	0.02 to 4.03	***R***^2^ = 0.06 Adjusted ***R***^2^ = -0.10 ***p*** = 0.75 **AIC** = -76.99
Fatigue	β1	-0.051	0.21	0.83	-0.55 to 0.45	
DOMS	β2	-0.12	0.45	0.65	-0.71 to 0.46	
PHV (years)	β3	-0.06	1.08	0.29	-0.1783 to 0.05799	
**TM**	β0	1.19	1.30	0.21	-0.75 to 3.15	***R***^2^ = 0.24 Adjusted ***R***^2^ = 0.10 ***p*** = 0.20 **AIC** = -76.60
Fatigue	β1	0.37	1.40	0.17	-0.18 to 0.93	
DOMS	β2	-0.01	0.22	0.82	-0.13 to 0.10	
PHV (years)	β3	-0.04	1.10	0.28	-0.13 to 0.04	
**TS**	β0	2276	0.32	0.75	-12,629 to 17,180	***R***^2^ = 0.18 Adjusted ***R***^2^ = 0.03 ***p*** = 0.33 **AIC** = 279.5
Fatigue	β1	-2175	1.22	0.23	-5932 to 1581	
DOMS	β2	378.8	0.18	0.85	-3994 to 4751	
PHV (years)	β3	430.1	1.04	0.31	-447.0 to 1307	

## Discussion

This study aimed to compare training load parameters between 1^st^ and 2^nd^ halves of the season and to analyze the relationships among those training load parameters with PHV, DOMS and fatigue status in U14 soccer players. The main results showed that TM was higher in the 2^nd^ half, while CW, AW and DOMS were higher in the 1^st^ half of the season. Moreover, there was a positive correlation between PHV and TS2 and between fatigue and TM (1^st^ half of the season).

Regarding TM, the present results pointed to a higher variation in the 2^nd^ half of the season, which the lower SD can explain. This result suggests that the training imposed in the 2^nd^ half of the season showed minor variations for all players, despite the different playing positions and the playing time, which was not considered in the present analysis. Nonetheless, a previous study did not find any significant difference among playing positions through the accumulated load, which supports the current analysis [14]. Moreover, it is relevant to note that both halves of the season showed values higher than 2 A.U. of monotony which has been considered a higher risk for non-contact injuries [28] or a higher risk for illness and overtraining [29], but it was not a case for the present study. In recent research that reviewed studies about young soccer players and training monotony, it was also concluded that monotony should not be used as a predictor of injuries [30]. This seems plausible considering that the present study achieved values of ~ 4–6 A.U. and even more if we believe data from professional soccer player that did not overcome 3.8 A.U. [31] or 7.2 A.U. [32]. This is why TM should be used to observe the weekly intensity variability, as Foster proposed [29].

Considering CW and AW, higher values were found in the 1^st^ half of the season, followed by higher values of DOMS compared to the importance of the 2^nd^ season. A previous study on U16 players also found higher values of ACWLR in the early season [33]. However, a prior survey of U16 players showed that higher values for CW were found at the season's end [34]. Similarly, other studies conducted with U16 soccer players presented higher values at the end season [34–36], which contradicts the current results. Nonetheless, such findings are not unique compared to data from professional players. For instance, a professional European soccer team also presented higher values in the 1^st^ half than in the 2^nd^ half for the ACWLR [32].

In addition, another study showed a significant correlation between CW and weekly DOMS (accumulated DOMS of the week) [36], which may help explain the present results, although no correlation was found in the present study. The different results could be associated with the different approaches used for data analysis. None of the two studies [34, 36] analyzed U14 soccer players, suggesting more studies with similar designs and larger sample sizes. Even in professional basketball players, higher values of DOMS and fatigue were found to be related to AW and ACWLR [37], but this was not shown in this study. Therefore, other contextual factors may influence the well-being of elite U14 soccer players (e.g., external load, nutritional intake, psychological and social aspects).

Another study in U16 soccer players found higher values of CW in the mid-season and lower values in the early season, while higher values of fatigue and DOMS were found at the end of the season, which is in opposition to the present results [12]. The same study [12] also found correlations between AW, TM and TS with well-being measures, while the present study failed to show such associations. Again, the differences between results could be explained by the different approaches for data analysis (accumulated values versus mean values) and age categories (U16 versus U14), respectively. Furthermore, such players' training characteristics could be other, making it impossible to justify the difference. Even so, an important insight is that each team has a specific scenario that should be considered when comparing with other teams' data. Therefore, it is suggested to provide more background knowledge about the context of each team analysis (such as training and match characteristics, and their internal and external load while controlling other variables, such as nutritional habits, psychological well-being and social aspects that can affect all interpretations).

Fatigue did not change across the season. Recent research on U15 soccer players showed that higher playing time values contributed to lower fatigue index values (measured by the seven repeated sprint tests) [38]. Despite the tools for measuring fatigue being different and the present study did not consider for analysis playing time, it seems that the coaches and their staff properly adjust the load for all players independently of their playing time through the season. Nonetheless, it is essential to highlight that Hooper Index was translated into Iranian, and all results should be interpreted with caution, considering they are more related to the Iranian competition.

Regarding correlation analysis, the present study only showed a correlation between TS2 and PHV which seems natural considering previous literature. For instance, previous research pointed out that PHV can occur around 14 years old [39]. Moreover, a study of U11-U15 soccer players found that peak aerobic performance, speed, and agility occurred during the PHV, specifically between 13 and 14 years [40]. This also may help justify the correlations between PHV and TS2 that the higher values of TM can also explain in the 2^nd^ half of the season. However, other studies found that PHV can occur later, between 15 and 16 years [15, 41]. Considering that these studies were conducted with small sample sizes, the results are always related to the sample analyzed and cannot be extrapolated for all athletes. It has been reported that achieving puberty first and, consequently, higher values of height is associated with the ability to run fast [42], which is also associated with a higher probability of success in achieving the professional status of a soccer player [43].

The present study presents some limitations. The small sample size of only one team from Iran avoids results generalizations. While this study addressed some wellness status through the Hooper index, namely, fatigue and DOMS, it failed to manage stress and sleep quality which should be considered in future research.

Moreover, playing positions analysis was not considered because playing positions in the present team changed through the season, which would limit the investigation. Additionally, previous studies highlighted that playing time [44] or playing status (i.e., starters versus non-starters) [12, 32] could have an impact on the training load. However, the present study failed to address such situations. Furthermore, training load parameters were only assessed by RPE, while other running and accelerometry-based variables could have strengthened the current research, as pointed out in previous work [34].

Despite the previous limitations, the strength of this study seems to be related to the lack of significant results when analyzing the relationship between wellness and training load in U14 soccer players, which also appears to be the first study to consider it. Thus, the main practical application to coaches and their staff is that internal training load and wellness parameters (i.e., DOMS and fatigue) should be applied simultaneously because they offer different players insights.

## Conclusions

In conclusion, there was a tendency for higher values in training load parameters in the 1^st^ half of the season. In addition, there were few associations between load parameters and wellness status. Finally, DOMS, fatigue status, and PHV did not explain variations in internal training loads.

## Data Availability

The data presented in this study are available on request from the corresponding author.

## Abbreviations

PHV: Peak Height Velocity
AW-Total: Total Amount of Acute Workload
CW-Total: Total Amount of Chronic Workload
ACWLR-Total: Total Amount of Acute: Chronic Workload Ration
TM-Total: Total Amount of Training Monotony
TS-Total: Total Amount of Training Strain
DOMS: Delayed Onset Muscle Soreness
